# Effects of Collection Durations on the Determination of Energy Values and Nutrient Digestibility of High-Fiber Diets in Growing Pigs by Total Fecal Collection Method

**DOI:** 10.3390/ani10020228

**Published:** 2020-01-31

**Authors:** Zhengqun Liu, Ruqing Zhong, Liang Chen, Fei Xie, Kai Li, Lei Liu, Hongfu Zhang

**Affiliations:** State Key Laboratory of Animal Nutrition, Institute of Animal Science, Chinese Academy of Agricultural Sciences, Beijing 100193, China; liuzhengqun2015@163.com (Z.L.); zhongruqing@caas.cn (R.Z.); xiefeihs@aliyun.com (F.X.); xndxlk@139.com (K.L.); swina2010@163.com (L.L.)

**Keywords:** collection duration, total fecal collection method, energy value, apparent total tract digestibility, high-fiber ingredient, growing pig

## Abstract

**Simple Summary:**

The total fecal collection method is the gold standard to estimate the energy values and nutrient digestibility of the swine diet. However, there is no standard collection duration for animals that should be sampled in swine research using the total fecal collection method. Thus, this study aimed to investigate the effects of different collection durations (3-day, 5-day, or 7-day) on energy values and nutrient digestibility of high-fiber diets in growing pigs by time-based total fecal collection method. The results showed that the digestible energy (DE), metabolizable energy (ME), and apparent total tract digestibility (ATTD) of gross energy (GE) and most nutrients in diets decreased linearly as the collection duration increased from a 3-day to a 7-day collection. However, there were no differences in the ATTD of GE and nutrient between the 5-day and 7-day collection durations. In addition, the energy values and the ATTD of GE and nutrient of high-fiber ingredients (sugar beet pulp (SBP) or defatted rice bran (DFRB)) were also not affected by the collection durations. Therefore, the results of this research suggest that a 5-day collection duration is adequate to determine the energy values and the ATTD of nutrient in diets containing high-fiber ingredients for growing pigs by time-based total fecal collection method.

**Abstract:**

This study was conducted to evaluate the effect of collection durations on the energy values and nutrient digestibility of high-fiber diets in growing pigs with a time-based total fecal collection method. A total of 24 barrows (body weight (BW): 31.1 ± 1.5 kg) were allotted to a completely randomized design with three diets. Diets included a corn–soybean meal (CSM) basal diet and two additional diets containing 20% sugar beet pulp (SBP) or defatted rice bran (DFRB) by replacing corn, soybean meal, and soybean oil in the CSM diet, respectively. Each diet was fed to eight barrows for a 7-day adaptation period followed by a 7-day total feces and urine collection period. The 7-day collection duration was divided into three collection phases, namely, phase 1 (days 8 to 11), phase 2 (days 11 to 13), and phase 3 (days 13 to 15). Then, similar portions of feces and urine from the different collection phases were composited into three additional samples (days 8 to 11, days 8 to 13, and days 8 to 15, respectively). The results showed that the digestible energy (DE), metabolizable energy (ME), and apparent total tract digestibility (ATTD) of gross energy (GE) and nutrient in experimental diets decreased linearly as the collection durations increased from a 3-day to a 7-day collection (*p* < 0.05). However, there were no differences in the energy values, GE, and nutrient digestibility of diets and of high-fiber ingredients between the 5-day and 7-day collection durations. In conclusion, this study suggests that a 5-day collection duration is adequate to determine the energy values and nutrient digestibility of high-fiber diets containing SBP or DFRB in growing pigs by the time-based total fecal collection method.

## 1. Introduction

The increasing usage of high-fiber ingredients in swine diets is thought to reduce the feed cost in swine production [1], improve body metabolism and intestinal health [2,3,4], contribute to the welfare of animals [5], and reduce the ammonia emissions from manure [6]. In contrast to these beneficial effects, high-fiber ingredients formulated in diets may also have a negative impact on the apparent total tract digestibility (ATTD) of gross energy (GE) and nutrient [7,8,9]. To take full advantage of these ingredients, therefore, the energy values and nutrient digestibility in high-fiber feed ingredients must be accurately estimated before they are used in the diet formulation.

The total fecal collection method is the gold standard to estimate the energy values and nutrient digestibility of swine diets [10]. Although the marker-to-marker procedure is preferred for fecal collection, the time-based feces collection procedure is inevitable in the studies, and usually a collection duration of four to six days for the marker-to-marker procedure was recommended [11,12,13,14]. Additionally, the time-based total fecal collection method is based on the assumption that, over an extended adaptation period, pigs can achieve a constant feed intake and feces output during the collection period [13,14]. However, the gastrointestinal tract emptying and digesta passage rate could not be kept constant because they could be affected by the environment, physiological, and health status of animals, as well as the physicochemical properties of the feed [15,16]. In particular, the source and level of dietary fiber in diet were important dietary factors that could affect the daily fecal excretion of pigs [16,17,18,19,20,21,22]. Therefore, an adequate collection duration is necessary to gather more representative samples for the energy value evaluation of high-fiber ingredients. However, there is no standard collection duration that should be sampled in swine research, and in recent studies, 3-day to 10-day collection durations have been used in swine nutrition and energy balance trials with the total fecal collection method [23,24,25,26].

Sugar beet pulp (SBP) and defatted rice bran (DFRB) are commonly used high-fiber dietary ingredients in swine diets, and they have different compositions and levels of total dietary fiber (TDF) [27,28,29]. To date, few experiments have directly compared the energy values and nutrient digestibility of high-fiber diets containing SBP or DFRB affected by different collection durations with the time-based total fecal collection method. Thus, the objectives of this study were to compare the different collection durations with time-based total fecal collection effects on estimating the energy values and ATTD of nutrient of the high-fiber diets containing SBP or DFRB in growing pigs.

## 2. Materials and Methods

The experimental protocol was approved by the Experimental Animal Welfare and Ethical Committee of the Institute of Animal Science, Chinese Academy of Agriculture Sciences (Ethics Approval Code: IAS2019-32).

### 2.1. Animals and Housing

A total of 24 crossbred barrows (Duroc × (Yorkshire × Landrace)) with initial body weight (BW) of 31.1 ± 1.5 kg were individually housed in stainless steel crates (1.2 by 1.5 m) equipped with a feeder and a nipple drinker. The crates had adjustable sides and were located in a room with temperature controlled at 25 °C ± 2.5 °C. Humidity varied from 55% to 65% during the experiment. An adjustable screen was placed under each crate that permitted the total collection of feces and urine.

### 2.2. Diets and Experimental Design

The chemical composition of corn, soybean meal, SBP, and DFRB in the experiment was analyzed (Table 1). Pigs were allotted a completely randomized design with 3 diets and 8 replications [30]. Diets included a corn–soybean meal (CSM) basal diet and 2 additional diets which were formulated by replacing corn, soybean meal, and soybean oil in the CSM diet with 20% SBP or DFRB, respectively (Table 2). Vitamins and minerals were supplemented in all diets to meet or exceed nutrient requirements of pigs according to the NRC (2012) [31]. All diets were fed in a mash form.

During the experimental periods, the daily feed allowance was calculated as 4% of the initial BW of each pig. Pigs were fed one-half of the daily feed allowance at 9:00 a.m. and 5:00 p.m. and provided ad libitum access to water. The initial 7 days were considered an adaptation period to the diet, followed by a 7-day total fecal and urine collection. The 7-day collection duration was divided into 3 collection phases, namely, phase 1 (days 8 to 11), phase 2 (days 11 to 13), and phase 3 (days 13 to 15). In each collection phase, a preservative of 50 mL of 6 N HCl was added to collection buckets placed under the metabolism crates. Feces and urine were collected and weighed, and all the feces and a 20% subsample of the urine were stored at −20 °C. Feces and urine from 3 different collection periods were kept separated and labeled accordingly; similar portions of feces and urine from the different collection periods were then composited into 3 additional samples (from days 8 to 11, days 8 to 13, and days 8 to 15) and labeled accordingly [32,33]. During the collection period, feed refusals and spillage were collected daily and subsequently dried and weighed [34].

### 2.3. Chemical Analysis

At the completion of the experiment, feces samples from the experiment were thawed and oven-dried at 65 °C. The ingredients of corn, soybean meal, SBP, and DFRB, together with 3 experimental diets were ground through a 0.5 mm screen in a centrifugal grinder before analysis.

Samples of ingredients, diets, and feces were analyzed for dry matter (DM) [35], crude protein (CP) [36], extract ether (EE; method 954.02; AOAC [37]), ash (method 942.05; AOAC [37]), TDF (method 991.43; AOAC [37]), and insoluble dietary fiber (IDF, method 991.43; AOAC [37]). The content of soluble dietary fiber (SDF, %) in the ingredients and diets was calculated according to the following equation (SDF = TDF − IDF). The organic matter (OM, %) content in the ingredients, diets, and feces was calculated as the difference between DM (%) and ash (%). The concentration of total carbohydrates (CHO, %) in the diets and feces was calculated according to the following equation (CHO = DM − CP − EE − ash) [38]. Neutral detergent fiber (NDF) and acid detergent fiber (ADF) were determined using filter bags and fiber analyzer equipment (Fiber Analyzer; ANKOM Technology, Macedon, NY, USA) following a modification of the procedures [39]. The content of GE in the feed ingredients, diets, feces, and urine samples was also analyzed using a bomb calorimeter (model 6400, Parr Instruments, Moline, IL, USA). All the analyses were performed in duplicate.

### 2.4. Calculations and Statistical Analysis

The digestible energy (DE), metabolizable energy (ME), and ATTD of GE and nutrient in the experimental diets were calculated according to the total fecal and urine collection method [13]. Additionally, the DE, ME, and ATTD of DM, GE, CP, and OM in SBP and DFRB were calculated by the difference method [13,40].

Data about the DE, ME, and ATTD of GE and nutrient in diets were analyzed by two-way ANOVA using the MIXED procedure of SAS (Version 9.4, SAS Institute, Cary, NC, USA). In the model, Diet, Duration, and interaction of Diet and Duration were fixed effects; Animal was a random effect. Least squares means were calculated and separated by the TDIFF option with Tukey’s adjustment [23,41,42]. Polynomial contrast was conducted to determine the linear and quadratic effects of the collection duration on the energy values and ATTD of GE and nutrient. Statistical significance was considered at *p* < 0.05, and a trend was considered if the *p*-value was between 0.05 and 0.10.

## 3. Results

### 3.1. Nutrient Composition of Ingredients and Diets

The nutrient composition values of the ingredients and experimental diets are shown in Table 1 and Table 2. The greatest GE content was found in soybean meal (4117 kcal/kg, as-fed basis), intermediate in corn (3841 kcal/kg, as-fed basis) and DFRB (3834 kcal/kg, as-fed basis), and the SBP (3623 kcal/kg, as-fed basis) had the least GE content. The concentrations of total CHO ranged from 38.17% in soybean meal to 73.04% in corn. The least concentrations of NDF and ADF were found in corn (8.89% and 1.74%, respectively), intermediate in soybean meal (22.00% and 8.77%, respectively) and DFRB (23.05% and 9.03%, respectively), and the SBP (38.56% and 21.49%, respectively) had the greatest NDF and ADF content. The concentrations of IDF for SBP and DFRB were 45.53% and 30.44%, respectively, and greater than those in corn and soybean meal. The SBP had the greatest SDF content, which was 16.15%, while the concentration of SDF in the other three ingredients ranged from 1.45% to 1.84%. Furthermore, the SDF/TDF ratio ranged from 6.5% in DFRB to 26.2% in SBP. It showed that SBP had numerically greater concentrations of NDF, ADF, TDF, IDF, and SDF than all other ingredients. Therefore, the SBP diet had numerically greater concentrations of NDF, ADF, TDF, IDF, and SDF than the CSM diet and the DFRB diet, as well. However, the SBP diet had similar GE content with the DFRB diet (3837 and 3854 kcal/kg as-fed basis, respectively).

### 3.2. Effect of Experimental Diets and Collection Duration on Energy Balance in Growing Pigs

All pigs remained healthy throughout the experiment and readily consumed their designated diets.

The fecal and urine output, energy excreted in feces and urine, DE and ME, and the ATTD of GE in the CSM, SBP, and the DFRB diets are presented in Table 3. No interaction of diet type and collection duration on the fecal output, urine output, DE, ME, and ATTD of GE was observed. The fecal output, energy excreted in feces and urine, DE, ME, and ATTD of GE in diets were significantly affected (*p* < 0.01) by the diet type. The fecal output was greatest in the DFRB diet (194.9 g DM/day), intermediate in the SBP diet (166.1 g DM/day), and least in the CSM diet (136.2 g DM/day, *p* < 0.05). Furthermore, the energy excreted in feces was least in the CSM diet (492.8 kcal/day), intermediate in the SBP diet (642.3 kcal/day), and greatest in the DFRB diet (708.6 kcal/day, *p* < 0.05). However, there was no difference in average daily energy excreted in feces between the SBP and DFRB diets. The energy excreted in the urine for pigs fed the CSM diet was 115.8 kcal/day, and greater than those in the SBP (88.6 kcal/day) and DFRB (98.7 kcal/day, *p* < 0.05) diets. The DE, ME, and ATTD of GE were greatest in the CSM diet (3501 kcal/kg as-fed basis, 3401 kcal/kg as-fed basis, and 89.08%, respectively), intermediate in the SBP diet (3257 kcal/kg as-fed basis, 3175 kcal/kg as-fed basis, and 84.89%, respectively), and least in the DFRB diet (3223 kcal/kg as-fed basis, 3137 kcal/kg as-fed basis, and 83.63%, respectively, *p* < 0.05). However, there were no differences in urine output among the three experimental diets.

The fecal DM output, urine output, and energy excreted in feces and in urine were not affected by the collection durations. The ME, DE, and ATTD of GE were affected by the collection durations, significantly or with a trend (*p* < 0.1). The DE, ME, and ATTD of GE decreased linearly from 3350 to 3309 kcal/kg (as-fed basis), 3261 to 3221 kcal/kg (as-fed basis), and 86.44% to 85.41%, respectively, as the collection duration increased from a 3-day to a 7-day collection (*p* < 0.05). However, there were no differences in DE, ME, and ATTD of GE in diets between the 5-day and 7-day collection durations.

### 3.3. Effect of Experimental Diets and Collection Duration on the ATTD of Nutrient in Diets

The ATTD values of nutrient in the experimental diets are presented in Table 4. There was no interaction of diet type and collection duration on the nutrient digestibility of diets. The ATTD of nutrient in experimental diets was significantly affected by the diet type (*p* < 0.01). The ATTD values of DM, OM, CHO, CP, and EE of the CSM diet (86.84%, 90.41%, 92.56%, 85.92%, and 82.88%, respectively) were higher than those in SBP (83.34%, 87.17%, 90.45%, 78.03%, and 76.78%, respectively, *p* < 0.05) and DFRB (80.64%, 85.32%, 87.18%, 80.81%, and 79.13%, respectively, *p* < 0.05) diets. Pigs fed the SBP diet had greater ATTD of DM, OM, and CHO than the DFRB diet. However, the ATTD of CP for pigs fed the SBP diet was lower than the DFRB diet (*p* < 0.05). The greatest ATTD of NDF and ADF was found in the SBP diet (79.30% and 81.42%, respectively), intermediate in the CSM diet (74.73% and 73.42%, respectively), and least in the DFRB diet (65.19% and 59.92%, respectively, *p* < 0.05).

The ATTD of DM, OM, EE, NDF, ADF, and ash in diets was significantly affected by the collection duration (*p* < 0.05). The ATTD of DM, OM, CP, EE, NDF, ADF, and ash in diets decreased linearly from 84.32% to 83.09%, 88.15% to 87.26%, 82.19% to 80.98%, 82.76% to 78.12%, 75.44% to 70.67%, 74.15% to 68.88%, and 43.78% to 38.85%, respectively, as the collection duration increased from a 3-day to a 7-day collection (*p* < 0.05). In addition, the ATTD of EE of the experimental diets was also quadratically decreased with the increasing collection durations (*p* < 0.05). However, there were no differences in the ATTD of CHO in diets determined with different collection durations. The ATTD of nutrient determined with a 3-day collection duration was higher than that determined with a 7-day collection duration (*p* < 0.05). However, there were no differences in ATTD of nutrient of diets between the 5-day and 7-day collection durations, except that the ATTD of NDF of diets determined with a 5-day collection duration was higher than the digestibility determined with a 7-day collection duration (*p* < 0.05).

### 3.4. Effect of Collection Duration on the Concentration of DE, ME, and ATTD of GE, DM, OM, CP, and CHO in High-Fiber Ingredients

As shown in Table 5, no differences of DE, ME, and ATTD of GE, DM, OM, CP, and CHO in SBP and DFRB among different collection durations were observed. Additionally, the determined DE and ME in SBP ranged from 2469 to 2499 kcal/kg (as-fed basis) and 2455 to 2482 kcal/kg (as-fed basis) among three collection duration treatments, respectively. The ATTD of GE, DM, OM, CP, and CHO of SBP ranged from 72.91% to 73.70%, 74.04% to 74.84%, 79.04% to 80.33%, 51.10% to 52.04%, and 85.72% to 89.30%, respectively. Furthermore, the determined DE and ME in DFRB ranged from 2310 to 2323 kcal/kg (as-fed basis) and 2265 to 2282 kcal/kg (as-fed basis), respectively. The ATTD of GE, DM, OM, CP, and CHO of DFRB ranged from 66.81% to 67.15%, 60.10% to 61.46%, 69.36% to 70.93%, 64.62% to 66.17%, and 70.18% to 71.56%, respectively.

## 4. Discussion

The high-fiber ingredients used in this experiment were SBP and DFRB, which have a big difference in dietary fiber content [31]. In the present experiment, the components of GE, TDF, IDF, and SDF in SBP and DFRB were within the range of reported content in these feed ingredients [19,31,43,44,45,46,47,48]. The results showed that a high-fiber ingredient (SBP and DFRB) formulated in diets could decrease the DE, ME, and ATTD of GE and most nutrients, and these results were consistent with previous studies [7,8,9,44,45]. However, in agreement with the studies of Zhang et al. [49], Zhang et al. [45], and Lyu et al. [43], SBP formulated in the diet could increase the ATTD of NDF and ADF for growing pigs. This probably occurred because SBP had a higher amount (26.2%) of SDF content (fermentable non-starch polysaccharides) in the TDF content, which can be easily fermented to short-chain fatty acids by hindgut microbes [6,49,50,51]. Compared with the DFRB diet, pigs fed the SBP diet had higher DE, ME, and most nutrient digestibility with the exception of ATTD of CP. These may be attributed to the differences in components and physical-chemical properties between the SBP and DFRB. For example, SBP had numerically lower digestible CP and greater digestible dietary fiber (SDF) concentrations than DFRB [27,31].

In the current experiment, the energy content and nutrient digestibility in diets decreased as the collection duration increased from a 3-day to a 7-day collection. These results may be due to the fact that the average daily amount of fecal DM output was not constant but increased from 159.0 to 170.9 g, as collection duration increased from a 3-day to a 7-day collection. Similarly, Li et al. [12] found that the amount of fecal collection by the time-based procedure was less than the amount of fecal collection by the marker-to-marker procedure for pigs fed the barley–canola meal diet. The assumption of using the total fecal collection method was that over an extended adaptation period, pigs could achieve a constant feed intake and fecal output during the collection period [13,14]. These results indicated the assumption may not be true. A possible reason for the fecal output not remaining constant might be that the rate of digesta passage in the gastrointestinal tract was not consistent but was pulsatile over time [15,52]. Furthermore, dietary fiber content and type of diet might be two of the reasons that influence the gastrointestinal emptying rate and the rate of digesta passage [16,17,18,19,53,54]. All these results indicated that a sufficient collection duration for the total fecal collection method is essential for a representative sample collection. In the current study, DE, ME, and ATTD of GE and nutrient in diets decreased as the collection duration increased from a 3-day to a 7-day collection, but there were no differences in determined DE, ME, and ATTD of GE and most nutrients of the experimental diets between the 5-day and 7-day collection durations. Therefore, the 5-day collection duration may be enough to collect representative samples to determine the energy and nutrient digestibility for pigs by time-based total fecal collection method [13].

The determined DE and ME values in SBP and in DFRB were within the range of reported values for these ingredients [19,31,43,44,45,47]. The results showed that there were no differences in DE, ME, and ATTD of GE, DM, CP, and CHO in SBP and DFRB among different collection durations with the time-based total fecal collection method, respectively. Similarly, Agudelo et al. [55] found that there no differences in nutrient digestibility of diets determined by total fecal collection method with the time-based or marker-to-marker procedure. Da Teixeira [11] and Wang and Adeola [23] found no differences in GE and nutrient digestibility of diets determined by total fecal collection method with different collection durations for pigs or small ruminants. Bakker and Jongbloed [25] also found that there was no influence in the recovery of the digestibility marker between the 3-day and 10-day total collection of feces; however, shortening the collection duration from a 10-day to a 3-day collection increased the standard deviations. In addition, a collection duration of at least 4 days of the total fecal method was recommended by Adeola et al. [13]. Therefore, to collect representative samples and decrease the standard deviations of the energy value determination, a 5-day collection duration is recommended to determine the energy and nutrient digestibility in high-fiber ingredients for pigs by time-based total fecal collection method.

## 5. Conclusions

In conclusion, the DE, ME, and ATTD of GE and most nutrients of the experimental diets decreased linearly as the collection duration increased from a 3-day to a 7-day collection. However, there were no differences in the energy content and nutrient digestibility in diets and in SBP or DFRB between the 5-day and 7-day collection durations. Therefore, this study suggests that a 5-day collection duration is adequate to determine the energy values and nutrient digestibility of high-fiber diets containing SBP or DFRB in growing pigs by the time-based total fecal collection method.

## Figures and Tables

**Table 1:** Analyzed chemical composition of ingredients (as-fed basis) ^1^.

Item	Corn	Soybean Meal	Sugar Beet Pulp	Defatted Rice Bran
Dry matter, %	87.46	89.54	93.39	91.06
Organic matter ^2^, %	86.14	83.50	83.11	80.53
Crude protein, %	7.67	42.15	9.58	16.80
Ether extract, %	5.43	3.18	2.80	3.34
Ash, %	1.32	6.04	10.28	10.53
Total carbohydrate ^2^, %	73.04	38.17	70.73	60.39
Neutral detergent fiber, %	8.89	16.43	38.56	23.05
Acid detergent fiber, %	1.74	5.36	21.49	9.63
TDF, %	11.18	16.94	61.68	32.02
IDF, %	9.73	15.10	45.53	30.44
SDF ^2^, %	1.45	1.84	16.15	1.58
SDF/TDF ratio, %	13.0	10.9	26.2	4.9
Gross energy, kcal/kg	3841	4117	3623	3834

^1 ^TDF, total dietary fiber; IDF, insoluble dietary fiber; SDF, soluble dietary fiber.^ 2^ Calculated value.

**Table 2:** Ingredients and analyzed nutrient compositions of the experimental diets (as-fed basis) ^1^.

Item	Diet		
	Basal	SBP	DFRB
Ingredients, %			
Corn	66.50	52.43	52.43
Soybean meal	25.00	19.71	19.71
Soybean oil	3.00	2.36	2.36
Sugar beet pulp	0.00	20.00	0.00
Defatted rice bran	0.00	0.00	20.00
Dicalcium phosphate	1.35	1.35	1.35
Limestone	0.75	0.75	0.75
Premix *	3.00	3.00	3.00
Salt	0.40	0.40	0.40
Total	100.00	100.00	100.00
Nutrient compositions			
Dry matter, %	89.61	90.18	89.72
Organic matter ^2^, %	82.61	82.42	81.32
Crude protein, %	15.95	14.63	15.71
Ether extract, %	7.53	6.47	6.38
Ash, %	7.00	7.75	8.40
Total carbohydrate ^2^, %	59.13	61.33	59.23
Neutral detergent fiber, %	11.39	15.66	13.55
Acid detergent fiber, %	3.62	7.25	4.90
TDF, %	14.54	24.43	18.71
IDF, %	12.81	20.66	17.49
SDF ^2^, %	1.73	3.77	1.22
SDF/TDF ratio, %	11.9	15.4	6.5
Gross energy, kcal/kg	3930	3837	3854

^1 ^SBP, sugar beet pulp; DFRB, defatted rice bran; TDF, total dietary fiber; IDF, insoluble dietary fiber; SDF, soluble dietary fiber. ^2^ Calculated value. * Provided the following quantities per kg of complete diet: vitamin A, 500 kIU; vitamin D3, 200 kIU; vitamin E, 1000 mg; vitamin K activity, 200 mg; vitamin B1, 150 mg; riboflavin, 400 mg; riboflavin, 3.52 mg; D-pantothenic acid, 1000 mg; niacin, 2000 mg; Cu (as copper chloride), 9 mg; I (as ethylenediamine dihydroiodide), 20 mg; Fe (as ferrous carbonate), 8000 mg; Mn (as manganese oxide), 4000 mg; and Zn (as zinc oxide), 4000 mg.

**Table 3:** Effects of collection duration on the energy value and gross energy digestibility for experimental diets fed to growing pigs ^1^.

Diet type	Collection Duration	n	Fecal Output, g DM/day	Energy Excreted in Feces, kcal/day	Urine Output, L/day	Energy Excreted in Urine, kcal/day	DE, kcal/kg as-fed basis	ME, kcal/kg as-fed basis	ATTD of GE, %
Basal	3-day	8	128.2	463.5	2.7	112.9	3528	3429	89.76
	5-day	8	138.3	499.6	3.0	116.6	3495	3393	88.92
	7-day	8	142.3	515.3	3.1	117.8	3481	3382	88.57
Sugar beet pulp	3-day	8	161.4	623.2	1.8	86.7	3277	3195	85.41
	5-day	8	166.1	641.7	2.0	90.0	3255	3172	84.84
	7-day	8	171.0	661.8	2.0	89.0	3238	3157	84.40
Defatted rice bran	3-day	8	187.5	686.2	2.5	92.9	3244	3159	84.16
	5-day	8	197.9	714.2	2.8	98.2	3217	3128	83.47
	7-day	8	199.4	725.5	3.1	105.1	3209	3123	83.25
Basal		24	136.2 ^c^	492.8 ^b^	2.9	115.8 ^a^	3501 ^a^	3401 ^a^	89.08 ^a^
Sugar beet pulp		24	166.1 ^b^	642.3 ^a^	1.9	88.6 ^b^	3257 ^b^	3175 ^b^	84.89 ^b^
Defatted rice bran		24	194.9 ^a^	708.6 ^a^	2.8	98.7 ^b^	3223 ^c^	3137 ^b^	83.63 ^c^
	3-day	24	159.0	591.0	2.3	97.5	3350 ^x^	3261	86.44 ^x^
	5-day	24	167.4	618.5	2.6	101.6	3322 ^x,y^	3231	85.74 ^x,y^
	7-day	24	170.9	634.2	2.7	104.0	3309 ^y^	3221	85.41 ^y^
SEM			4.6	17.3	0.2	2.9	15.6	15.6	0.31
*p*-Value									
Diet			<0.001	<0.001	0.136	<0.001	<0.001	<0.001	<0.001
Duration			0.424	0.467	0.758	0.634	0.014	0.077	0.013
Diet × Duration			0.999	1.000	0.997	0.980	0.995	0.998	0.995
Linear			0.205	0.225	0.347	0.461	0.004	0.030	0.004
Quadratic			0.760	0.846	0.885	0.935	0.554	0.529	0.554

^1^ GE, gross energy; DE, digestible energy; ME, metabolizable energy; ATTD, apparent total tract digestibility; SEM, standard error of the mean.^ a,b,c^ Means for DE, ME, and ATTD of GE of the basal diet, sugar beet pulp diet, and defatted rice bran diet with different superscript letters were significantly different (*p *< 0.05).^ x,y^ Means for the collection duration on the concentration of DE, ME, and ATTD of GE of diets with different superscript letters were significantly different (*p *< 0.05).

**Table 4:** Effect of collection duration on nutrient digestibility for experimental diets fed to growing pigs ^1^.

Diet Type	Collection Duration	n	Apparent Total Tract Digestibility, %							
			DM	OM	CHO	CP	EE	NDF	ADF	Ash
Basal	3-day	8	87.65	90.99	92.92	86.71	84.87	76.56	74.91	48.34
	5-day	8	86.64	90.24	92.47	85.88	81.97	74.72	74.11	44.19
	7-day	8	86.23	89.99	92.33	85.18	81.80	72.91	71.25	41.87
SBP	3-day	8	83.91	87.54	90.40	78.58	80.71	81.15	83.41	45.38
	5-day	8	83.27	87.21	90.76	78.11	74.11	79.60	81.88	41.45
	7-day	8	82.83	86.77	90.19	77.39	75.51	77.16	78.96	40.99
DFRB	3-day	8	81.39	85.91	87.49	81.28	82.71	68.60	64.12	37.61
	5-day	8	80.33	85.02	86.94	80.77	77.63	65.02	59.21	34.91
	7-day	8	80.21	85.02	87.10	80.38	77.06	61.94	56.43	33.68
Basal		24	86.84 ^a^	90.41 ^a^	92.56 ^a^	85.92 ^a^	82.88 ^a^	74.73 ^b^	73.42 ^b^	44.80 ^a^
SBP		24	83.34 ^b^	87.17 ^b^	90.45 ^b^	78.03 ^c^	76.78 ^b^	79.30 ^a^	81.42 ^a^	42.61 ^a^
DFRB		24	80.64 ^c^	85.32 ^c^	87.18 ^c^	80.81 ^b^	79.13 ^b^	65.19 ^c^	59.92 ^c^	35.40 ^b^
	3-day	24	84.32 ^x^	88.15 ^x^	90.27	82.19	82.76 ^x^	75.44 ^x^	74.15 ^x^	43.78 ^x^
	5-day	24	83.42 ^y^	87.49 ^x,y^	90.06	81.59	77.90 ^y^	73.11 ^x^	71.73 ^x,y^	40.18 ^y^
	7-day	24	83.09 ^y^	87.26 ^y^	89.88	80.98	78.12 ^y^	70.67 ^y^	68.88 ^y^	38.85 ^y^
SEM			0.33	0.28	0.28	0.45	0.55	0.83	1.20	0.68
*p*-Value										
Diet			<0.001	<0.001	<0.001	<0.001	<0.001	<0.001	<0.001	<0.001
Duration			0.001	0.011	0.320	0.131	<0.001	<0.001	0.002	<0.001
Diet × Duration			0.974	0.943	0.581	0.994	0.571	0.757	0.712	0.878
Linear			<0.001	0.004	0.133	0.045	<0.001	<0.001	<0.001	<0.001
Quadratic			0.308	0.408	0.951	0.999	0.004	0.946	0.856	0.247

^1^ SBP, sugar beet pulp; DFRB, defatted rice bran; DM, dry matter; OM, organic matter; CHO, total carbohydrate; CP, crude protein; EE, ether extract; NDF, neutral detergent fiber; ADF, acid detergent fiber; SEM, standard error of the mean.^ a,b,c^ Means for ATTD of nutrient of the basal diet, sugar beet pulp diet, and defatted rice bran diet with different superscript letters were significantly different (*p *< 0.05).^ x,y^ Means for the collection duration on the ATTD of nutrient of diets with different superscript letters were significantly different (*p *< 0.05).

**Table 5:** Effect of collection duration on energy content, gross energy (GE), and nutrient digestibility of high-fiber ingredients fed to growing pigs ^1^.

Item		n	Collection Duration			SEM	*p*-Value	
			3-day	5-day	7-day		Linear	Quadratic
SBP	DE, kcal/kg as-fed basis	8	2478	2499	2469	34.45	0.928	0.750
	ME, kcal/kg as-fed basis	8	2461	2482	2455	42.23	0.959	0.799
	ATTD of GE, %	8	73.23	73.70	72.91	0.90	0.890	0.758
	ATTD of DM, %	8	74.04	74.84	74.26	0.83	0.923	0.718
	ATTD of OM, %	8	79.04	80.33	79.12	0.77	0.969	0.477
	ATTD of CP, %	8	51.10	52.04	51.22	1.68	0.979	0.821
	ATTD of CHO, %	8	85.72	89.30	87.01	0.68	0.430	0.047
DFRB	DE, kcal/kg as-fed basis	8	2313	2310	2323	34.61	0.913	0.913
	ME, kcal/kg as-fed basis	8	2280	2265	2282	57.40	0.992	0.902
	ATTD of GE, %	8	66.99	66.81	67.15	0.90	0.944	0.901
	ATTD of DM, %	8	61.46	60.10	61.16	0.97	0.907	0.587
	ATTD of OM, %	8	70.93	69.36	70.36	0.78	0.782	0.473
	ATTD of CP, %	8	64.62	65.32	66.17	1.70	0.729	0.985
	ATTD of CHO, %	8	71.18	70.18	71.56	0.76	0.850	0.496

^1^ SBP, sugar beet pulp; DFRB, defatted rice bran; DE, digestible energy; ME, metabolizable energy; ATTD, apparent total tract digestibility; DM, dry matter; GE, gross energy; CP, crude protein; OM, organic matter; CHO, total carbohydrates; SEM, standard error of the mean.
